# Advances in Porous Lightweight Materials and Lattice Structures

**DOI:** 10.3390/ma19142975

**Published:** 2026-07-10

**Authors:** Girolamo Costanza, Maria Elisa Tata

**Affiliations:** Industrial Engineering Department, University of Rome Tor Vergata, Via del Politecnico, 1-00133 Rome, Italy; elisa.tata@uniroma2.it

The demand for lightweight structures that ensure high mechanical strength and better overall performance is constantly growing in many engineering fields [1,2,3]. Studying the mechanical behavior of metallic and non-metallic porous materials, both under static and dynamic conditions, is becoming increasingly important. The thermal performance and heat transfer characteristics of phase change material–metal foam composites are key features in applications exploiting the thermophysical properties of PCM. The biocompatibility of metallic bone lattice structures is fundamental in the development of titanium artificial bones manufactured by additive manufacturing. This Special Issue (SI), “Advances in Porous Lightweight Materials and Lattice Structures”, presents recent experimental and simulation research findings regarding the mechanical behavior of porous and lightweight structures. This Editorial summarizes the six publications (research articles) included in the SI.

The first study examines the thermal performance of phase change material (PCM)–aluminum foam composites under base heating, finding that the inclusion of metal foam significantly reduces thermal lag and improves temperature uniformity compared to pure PCM in thermal energy storage and electronics cooling [4]. In the following, three key findings are reported:

Pore Density (PPI): Varying the pore density from 10 to 40 PPI at a constant porosity has negligible influence on melting time, as thermal enhancement is primarily governed by the conductive pathways of the metallic fraction [5].

Dominant Parameters: Heat flux is the most critical factor in melting speed, though it shows diminishing returns at higher levels. Additionally, PCMs with higher latent heat and melting temperatures (e.g., RT64HC) require significantly longer melting times.

Experimental Discrepancies: Existing empirical models for side-heated foams underpredict melting completion times for base-heated setups, largely due to unavoidable heat losses (3–15%) in the experimental apparatus [6].

In the second study, the researchers developed a modified Charpy impact machine to directly and accurately measure the energy and impact absorption of lattice structures. By using a piezoelectric accelerometer and the machine’s graduated display, this setup records data instantaneously, avoiding the 30–100% inaccuracies caused by complex post-processing in traditional methods [7]. Testing with a specialized sandwich specimen and a custom striker cape revealed that parallel gradient lattices can absorb energy at lower impact levels than uniform structures, while series gradients exhibit the highest impact [8]. This method offers a more reliable and efficient way to evaluate protective materials under high-strain-rate dynamic conditions.

The third study analyzes eight stent-graft configurations to optimize thoracic aortic repair through material selection [9]. Key findings indicate that PET grafts significantly enhance flexibility and fatigue resistance compared to e-PTFE [10]. Conversely, radial force (crimpability) is almost entirely dictated by the nitinol stent’s properties, with the choice of graft material having a negligible impact. Ultimately, pairing a nitinol stent with a low Young’s modulus and a PET graft (specifically Model MP-5) provides the optimal balance of flexibility and durability.

The fourth study introduces a fractal tree-inspired lattice core (SSFL) for sandwich structures, designed using symmetric branching patterns and manufactured via 3D printing [11]. Key findings include the following: multiple load-bearing (the fractal geometry allows for secondary and tertiary bearing stages, where branches take over the load after the main “trunk” fails) [12], superior energy absorption (the SSFL-2 design absorbs 10.6 times more energy than traditional pyramidal lattice structures), controllable deformation (the branching design shortens strut lengths, preventing brittle buckling and favoring more stable bending and fracturing mechanisms), and geometric sensitivity (performance is highly dependent on the cross-section dimensions and the bifurcation position). Finally, fractal design provides a robust strategy for creating high-performance, energy-absorbing engineering materials.

In the fifth study, researchers used the lost-PLA technique [13] to produce AA6082 aluminum alloy cellular structures with Kelvin cell geometry. This cost-effective method involves 3D printing a PLA model, creating a plaster mold, and casting the molten alloy to create high-accuracy replicas [14]. Mechanical tests revealed predictable compression behavior: an initial linear stage, two successive plateaus caused by plastic collapse and ligament buckling, and final densification. The foams achieved an average specific absorbed energy of 2.3 J/cm^3^, confirming the technique’s reliability for manufacturing complex, energy-absorbing metallic structures for engineering applications.

Finally, in the sixth study, researchers successfully evaluated the biocompatibility of a titanium artificial bone featuring a Body Diagonals with Nodes (BDN) lattice structure for mandibular reconstruction [15]. In a study using rabbit tibial defects, the BDN structure was found to be safe and effective, showing no signs of inflammation or implant failure over four weeks [16]. New bone and connective tissue integrated into the lattice, with the BDN structure producing more trabecular bone-like tissue than the comparison group. Because it combines high mechanical strength to withstand biting forces with excellent biological affinity, researchers concluded that a BDN lattice with a 3.5 mm cell size is the optimal design for future clinical use in jaw reconstruction.
